# Cardiometabolic Biomarkers and Habitual Caffeine Consumption Associate with the Adverse Ambulatory Blood Pressure Response to Strenuous Physical Exertion among Firefighters

**DOI:** 10.3390/nu14194025

**Published:** 2022-09-28

**Authors:** Rachel S. Berkowsky, Amanda L. Zaleski, Beth A. Taylor, Ming-Hui Chen, Kim M. Gans, Yin Wu, Paul M. Parducci, Yiming Zhang, Antonio B. Fernandez, Linda S. Pescatello

**Affiliations:** 1 Department of Kinesiology, University of Connecticut, Storrs, CT 06268, USA; 2 Department of Preventive Cardiology, Hartford Hospital, Hartford, CT 06102, USA; 3 Institute for Collaboration on Health, Intervention, and Policy, University of Connecticut, Storrs, CT 06268, USA; 4 Department of Statistics, University of Connecticut, Storrs, CT 06268, USA; 5 Department of Human Development and Family Studies, University of Connecticut, Storrs, CT 06268, USA

**Keywords:** acute cardiac event, aerobic exercise, hypertension, nutrition, physical activity, strenuous occupational hazard, vigorous exercise

## Abstract

Caffeine has beneficial effects on firefighter job performance reducing fatigue and improving psychomotor vigilance. However, excessive caffeine intake may raise blood pressure (BP) following a bout of acute exercise among adults with elevated BP. The influence of caffeine intake on the ambulatory BP (ABP) response to vigorous physical exertion among firefighters has not been studied. In this sub-study we conducted secondary statistical analyses from a larger clinical trial (NCT04514354) that included examining the influence of habitual caffeine intake, and cardiometabolic biomarkers shown to influence BP, on the ABP response following a bout of sudden vigorous exertion over 19 h among firefighters. Previously, we found high amounts of calcium and sodium intake raised BP following a bout of acute exercise among adults with elevated BP. Thus, other secondary aims were to examine the influence of habitual calcium and sodium intake, and cardiometabolic biomarkers have shown to influence BP, on the ABP response following sudden vigorous exertion over 19 h among firefighters. Firefighters (*n* = 15) completed a Food-Frequency Questionnaire assessing habitual dietary intake over the past year. They randomly completed a maximal graded exercise stress test (GEST) and non-exercise CONTROL on separate non-workdays leaving the laboratory wearing an ABP monitor for 19 h. Prior to and immediately after the GEST, fasting venous blood was collected to measure lipid-lipoproteins, c-reactive protein, and blood glucose. Height and weight were taken to calculate body mass index. Repeated measures ANCOVA tested if the ABP response differed after GEST vs. CONTROL. Linear mixed models examined the relationships among caffeine, calcium, sodium, cardiometabolic biomarkers, and the ABP response following GEST vs. CONTROL. Firefighters were middle-aged (40.2 ± 9.5 year), overweight (29.0 ± 3.9 kg/m^2^) men with elevated BP (124.1 ± 10.3/79.6 ± 11.5 mmHg) who consumed 542.0 ± 348.9 mg of caffeine/day, about ~50% more than the dietary reference intake. Unexpectedly, systolic ABP was higher by 18.0 ± 6.7 mmHg and diastolic ABP by 9.1 ± 5.4 mmHg (*p*s < 0.01) over 19 h following GEST vs. CONTROL. We found 24% of the variance in the adverse ABP response to maximal physical exertion was explained by caffeine intake, and when combined with c-reactive protein, non-high-density lipoprotein-cholesterol, body mass index, blood glucose, and resting heart rate, up to 74% of the variability in the ABP response was explained. Additionally, we found calcium (*p*s < 0.001) and sodium (*p* < 0.0001) intake each explained up to 24% of the ABP response. Further investigation is needed in a larger, more diverse sample of firefighters to better establish how caffeine contributes to the adverse BP response to strenuous physical exertion.

## 1. Introduction

Firefighters in the United States have a high prevalence of cardiovascular disease (CVD) and its risk factors, as 75% of firefighters have high blood pressure (BP) and 70% of firefighters are overweight to obese [1,2]. Furthermore, 61% of firefighters are physically unfit [3]. The prevalence of CVD and its risk factors are thought to contribute to the disproportionate incidence of sudden cardiac death among firefighters, which accounts for almost 50% of on-duty related deaths [4,5,6]. Of note, firefighters have a four-fold higher relative risk of sudden cardiac death on the job compared to the general population [1,7]. In the 2021 National Fire Service Research Agenda Report approximately 300 research recommendations having the most significant impact on firefighter wellbeing were made [8]. These recommendations included improving their overall health and safety by conducting research relating to cardiovascular health, hypertension, overexertion, and how diet, specifically caffeine and energy drinks, affect health and wellness among firefighters.

Postexercise hypotension (PEH) is the reduction in BP of 5–8 mmHg that immediately occurs following exercise among physically unfit and fit adults persisting for up to 24 h [9,10,11,12]. PEH is clinically meaningful for those with elevated BP because BP is reduced throughout the day when BP is typically at its highest levels. In fact, due to PEH, BP can become normalized after exercise for up to 24 h for some individuals with hypertension. The magnitude of PEH is directly proportional to exercise intensity, with the greatest BP reductions occurring after vigorous intensity exercise [11]. For these reasons, it appears PEH may have cardioprotective effects for firefighters on and off the job [13].

Career firefighters are more likely to consume fast foods, sugary drinks, and other less healthy meal choices due to the unpredictable nature of the occupation [1]. How diet may affect PEH among firefighters is not known, but there is suggestive evidence high caffeine intake can adversely affect PEH in healthy adults with high BP. Sarmento et al. found three cups of 1.2% caffeinated coffee completely abolished PEH after a bout of acute submaximal exercise among a small sample of adults with controlled hypertension [14]. Furthermore, Notarius et al. found an intravenous caffeine infusion of 4 mg/kg attenuated PEH compared to placebo after a bout of acute maximal aerobic exercise in a sample of middle-aged adults with elevated resting BP [15]. In this sub-study we conducted secondary statistical analyses from a larger clinical trial (NCT04514354) that included examining the influence of habitual caffeine intake, and cardiometabolic biomarkers shown to influence BP [9,11,16,17,18], on ambulatory BP (ABP) following a bout of sudden vigorous intensity exercise over 19 h among firefighters. We hypothesized high habitual caffeine intake would adversely influence the ABP response to sudden vigorous exertion compared to control among firefighters.

Previously we examined the influence of dietary calcium and sodium intake on PEH [16,17,18]. Pescatello et al. found a calcium intake of 1380 mg/day elicited PEH to lesser levels than consumption of lower amounts of calcium among 50 overweight men with hypertension [16]. Babcock et al. found consuming a sodium capsule containing 3900 mg of sodium for 10 days abolished PEH compared to a placebo dextrose capsule among 19 healthy subjects with normal to elevated BP [17]. Thus, other secondary aims of this sub-study were to examine the influence of habitual calcium and sodium intake, and cardiometabolic biomarkers shown to influence BP [9,11,16,17,18], on ABP following a bout of sudden vigorous exercise over 19 h among firefighters. We hypothesized high habitual calcium and sodium intake would adversely influence the ABP response to sudden vigorous exertion compared to control among firefighters.

## 2. Materials and Methods

### 2.1. Study Overview

**Figure 1:**
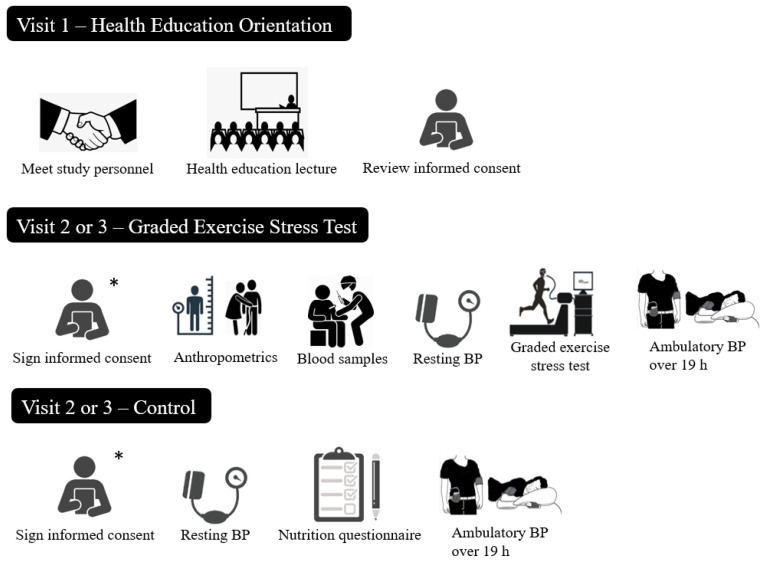
Study Design Overview. Note: BP = Blood pressure. * Subjects signed the informed consent during their Visit 2.

The present sub-study is part of a larger clinical trial titled, The Influence of Cardiorespiratory Fitness on Firefighter Cardiovascular Health Under Conditions of Heavy Physical Exertion (FIT and FIRED UP). FIT and FIRED UP was approved by the Institutional Review Boards of the University of Connecticut and Hartford Hospital with the clinical trial identifier NCT04514354. The subjects indicated their willingness to participate by signing informed consent. This sub-study examined the influence of dietary intake on resting BP and the ABP response following a maximal cardiopulmonary graded exercise stress test (GEST) compared to a non-exercise control day (CONTROL) among a subsample of 15 male firefighters. All three study visits were performed by the same trained investigator on non-workdays with start times prior to 10:00 am. Subjects were instructed to refrain from exercise, caffeine, alcohol, and smoking 24 h prior to each visit. Of note, there were no smokers in this clinical trial. The study design overview is shown in Figure 1.

### 2.2. Subjects

Subjects were fully active-duty firefighters employed by a local fire department in central CT. They were excluded if they were not able to comply with all study procedures, or if an injury or illness occurred that would not allow them to participate. Following the Joint National Committee 7th (JNC VII) BP classification scheme, subjects with a resting systolic BP (SBP) ≥ 160 and/or diastolic BP (DBP) ≥ 100 mmHg were also excluded [19]. Of note, the JNC VII BP classification scheme was the scheme available at the time of study data collection, and thus, these BP cutoffs were those we adhered to [19]. In addition, subjects confirmed they were taking the same medication for four weeks prior to Visit 1 and continued taking that medication throughout the study.

### 2.3. Study Design

#### 2.3.1. Visit 1: Health Education Orientation Session

Firefighters were invited to a health education orientation session, which was Visit 1. The purpose of Visit 1 was to familiarize the subjects with study personnel and procedures and to deliver a health education lecture on physical activity and “heart healthy” habits. Potential subjects received a sign-up sheet or expressed interest in the study via their personal email. They also received copies of the informed consent for review. Subjects reviewed and signed the informed consent at the start of the next study visit.

#### 2.3.2. Visit 2 or 3: Cardiovascular Health Testing

Subjects completed the cardiovascular health testing experiments (a maximal GEST or CONTROL) in random order on non-workdays that were separated by >24 h at the Department of Preventive Cardiology at Hartford Hospital, CT. Visits 2 and 3 were randomized to account for the circadian variation in ABP using www.randomization.com (accessed on 1 March 2015) [20]. All subjects were instructed to fast overnight for 10 h prior to Visits 2 and 3 and come to the laboratory well hydrated. They drank water ad libitum during these visits. Participants were asked to adhere to their usual dietary patterns during Visits 2 and 3.

*Maximal Cardiopulmonary Graded Exercise Stress Test*

On either Visit 2 or 3, subjects performed a maximal cardiopulmonary GEST using the Balke protocol [21]. Immediately prior to the GEST, the study physician or his designee performed a brief physical examination in which BP was assessed and relevant medical information (i.e., medical history, medications, family history) was reviewed. The physician who completed the physical examination remained present during the GEST to monitor the subjects’ electrocardiogram and other physical signs. After the GEST was completed, the study physician reviewed the electrocardiogram for signs of ischemia. We determined peak oxygen uptake (VO_2_peak) by breath-by-breath analysis of expired gases (i.e., oxygen and carbon dioxide) (ParvoMedicsTrueOne^®^ 2400 Metabolic Measurement System, ParvoMedics Inc., Sandy, UT, USA). We measured heart rate continuously with a 12-lead electrocardiogram system, and BP by auscultation every 3 min during the GEST. We gave a copy of the electrocardiogram to the subjects to share with their primary care provider.

Additional measures assessed during this visit included body composition, resting BP, and fasting blood samples pre- and post-GEST. The order in which these assessments took place were; Prior to the GEST: (1) body composition, (2) fasting blood samples, and (3) BP; and Immediately Post-GEST: (1) fasting blood samples, and (2) BP. Prior to leaving the laboratory, we attached an ABP monitor to the subject’s non-dominant arm as described below. The GEST Visit on average lasted about 1.5–2 h.

*Control*

During CONTROL, resting BP was measured using the same procedures as during the GEST Visit. At the conclusion of CONTROL, we fitted the subjects with the same ABP monitor following the same procedures as during the GEST Visit. In addition, subjects completed a food-frequency questionnaire. The CONTROL Visit lasted an average of 1 h. All methods are described below.

### 2.4. Study Procedures

#### 2.4.1. Dietary Intake—Food-Frequency Questionnaire

To assess usual dietary and alcohol intake over the past 12 months, our subjects completed the National Health and Nutrition Examination Survey (NHANES) semi-quantitative Food-Frequency Questionnaire [22] during CONTROL. This Food-Frequency Questionnaire is a validated tool for measuring the intake of macro- and micro-nutrients based on the average daily consumption of 127 food items [22,23,24,25]. These data were analyzed utilizing the National Cancer Institute Diet*Calc software v1.4.3 (Bethesda, MD) to produce daily food frequency estimates of the 127 nutrient and dietary constituents. The Database Utility converted nutrient values expressed as nutrients per 100 g into the nutrient per serving size format required by the Diet*Calc software [22,23,24,25]. Of note, the NHANES Food-Frequency Questionnaire measured all forms of caffeine consumption, e.g., coffee, energy drinks, iced tea, etc.) versus coffee consumption alone.

#### 2.4.2. Dietary Approaches to Stop Hypertension

Because the Dietary Approaches to Stop Hypertension (DASH) diet is recommended to treat hypertension [26,27], we calculated the DASH accordance score from the NHANES Food-Frequency Questionnaire data. The nine nutrient components comprising the DASH accordance score were sodium, cholesterol, saturated fat, total fat, protein, calcium, magnesium, potassium, and fiber [26]. Meeting the DASH target value for each nutrient was scored with 1 point, meeting the intermediate range value was scored with 0.5 points, and not meeting either target was scored with 0 points [26]. The score for each nutrient was summed to determine the overall DASH accordance score [26]. The DASH score ranges from 0 to 9, with a higher score indicating the subject was more DASH accordant. Subjects meeting half of the DASH target values of >4.5 were considered DASH accordant [27].

#### 2.4.3. Anthropometric Measurements

During the GEST Visit, height and weight were measured with a calibrated balance beam scale to calculate body mass index (BMI). Waist circumference was measured in duplicate at the iliac crest using a non-distensible Gulick tape measure.

#### 2.4.4. Resting Blood Pressure

During Visits 2 and 3, resting BP was measured according to the American Heart Association standards using an automated BPTRU monitor (BPTRU Medical Devices, Coquitlam, British Columbia, Canada) [28]. Subjects sat for 5 min and then BP was measured three times, 1 min apart in each arm. If the readings obtained were within 5 mmHg, these readings were averaged and recorded as resting BP. If these readings did not agree within 5 mmHg, up to three additional readings were taken. Of these, the three readings that agreed within 5 mmHg were averaged and recorded as resting BP. If there were not three readings that agreed within 5 mmHg, the three closest readings were averaged and recorded as resting BP.

#### 2.4.5. Blood Sampling and Analysis

Prior to and immediately after the GEST, a trained phlebotomist collected five tubes of venous blood (52 cc or 3.5 tablespoons) from the antecubital vein of each subject using a 21-gauge butterfly needle for the determination of fasting lipid-lipoproteins, glucose, insulin, high sensitivity C-reactive protein (hs-CRP), fibrinogen, cystatin C, and renin (Quest Diagnostics, LLC, Marlborough, MA, USA). Serum samples were archived in a locked −80 °C freezer at Hartford Hospital. All samples were sent to Quest Diagnostics^®^ to be analyzed in batch. Briefly, colorimetric enzymatic assays determined serum total cholesterol, triglycerides, high-density lipoprotein cholesterol (HDL-C), and low-density lipoprotein cholesterol (LDL-C) levels. Enzymatic/spectrophotometric methods and radioimmunoassay determined serum glucose and insulin levels, respectively. Immunoturbidimetry determined serum hs-CRP levels. Photometric clot formation measured plasma fibrinogen in duplicate. Particle enhanced turbidimetric immunoassay determined Cystatin C levels. Liquid chromatography/mass spectrometry measured plasma renin levels.

Blood sample results were reported according to Clinical Laboratory Improvement Amendments standards with each analyte’s coefficient of variation below the total error allowable criteria set forth by national criteria [29]. The coefficient of variations for the various biomarkers were: total cholesterol = 0.0175, triglycerides = 0.0190, HDL-C = 0.0274, LDL-C = 0.04, glucose = 0.0214, insulin = 0.0402, hs-CRP = 0.4, fibrinogen = 0.035, cystatin C = 0.10, and renin = 0.10. The Quest Diagnostics^®^ account manager sent results to the research staff in an encrypted Microsoft Excel file.

#### 2.4.6. Ambulatory Blood Pressure

The subjects were attached to an Oscar2 automatic noninvasive ABP monitor (Suntech Medical Instruments Inc., Raleigh, NC, USA) at the conclusion of the GEST and CONTROL. The same ABP monitor was worn by the same subject during Visits 2 and 3. A calibration check was done with a mercury sphygmomanometer using a t-tubule. The ABP monitor was programmed to record BP at regular intervals three times per waking hour and two times per sleeping hour. Subjects were instructed to proceed with normal activities, not to exercise, and to keep their arm still and extended at their side while each ABP measurement was taken. Subjects carried a standard journal, recording activities performed during each measurement, any unusual physical or emotional events, and sleep and wake times. The following morning subjects detached the monitor and returned it to the study investigators that day. If subjects were called into work during a study visit, they were instructed to remove the ABP monitor and discontinue their participation in the study for that day and repeat that study visit on a different day (*n* = 1). The average ABP monitor attachment time for CONTROL was 8:00 am and 10:30 am for the GEST.

Computerized ABP reports were considered acceptable if at least 80% of the BP readings were obtained. ABP readings >220 for SBP and/or >130 mmHg for DBP were omitted according to the manufacturer’s exclusion criteria. The ABP data were imported for analysis using the Accuwin Pro v3 software.

### 2.5. Statistical Analyses

Baseline descriptive characteristics are reported as mean ± standard deviation for the total sample. The Shapiro–Wilks tested if the data were normally distributed. An ABP change response variable was calculated at each hourly interval by subtracting the average ABP value at each hour from baseline BP over 19 h. To handle any missing ABP data, we utilized the full information maximum likelihood method to estimate model parameters. This method does not require missing values be replaced or imputed, but that missing data are handled within the analysis model. Thus, all available information can be used to estimate the model parameters [30].

In this sub-study, we conducted secondary statistical analyses from a larger clinical trial (NCT04514354) that included a repeated measures analysis of covariance (RMANCOVA) to test if the ABP response differed between conditions (GEST versus CONTROL) over the same hourly intervals among the total sample with resting BP as a covariate over 19 h. Age, BMI, waist circumference, VO_2_peak, and the DASH accordance score were entered as covariates but were found not to be significant. Simple linear regressions tested the correlations between ABP and the dietary factors and between resting BP and the dietary factors. Multivariate linear regressions examined relationships among resting BP and cardiometabolic biomarkers. In addition, linear mixed models examined the relationships among the ABP response following GEST versus CONTROL, the dietary factors, and the cardiometabolic biomarkers. Of note, in the linear mixed models and multivariate linear regressions, the individual partial portion of variance explained by each independent variable added up to greater than the overall portion of variance explained, which suggests the individual independent variables are all important in explaining the variation of the dependent outcome variable [31].

Post hoc power analyses were conducted for the linear mixed models using a simulation-based approach [32]. For each of the models, we simulated 50,000 independent sets of ABP responses over 19 h based on the observed predictors from the data and the estimated linear mixed model. In each of the simulations, the fixed effects were generated from a multivariate normal distribution where the mean vector is the estimated fixed effects in the linear mixed model, and the covariance matrix is the covariance matrix of the fixed effect estimates. The empirical statistical power for each of the predictors was calculated by the proportion of the significant results in the 50,000 simulations. The multiple hypothesis tests in each simulation were adjusted by the Holm–Bonferroni correction [33]. Variance inflation factors below 5.0 indicated there was minimal overlap among the independent variables in the multivariate linear regressions and linear mixed models [34]. Baseline descriptive analyses, Shapiro–Wilks, simple linear regressions, and multivariate linear regressions for resting BP were performed in SPSS Statistics version 25.0 (IBM Corporation, New York, NY, USA). The simple linear regressions, RMANCOVA, linear mixed models, and post hoc power analyses for ABP were performed in SAS 9.4 (Cary, NC, USA) and R 4.1.0 (R Foundation for Statistical Computing, Vienna, Austria). In all cases *p* < 0.05 was established as the level of statistical significance.

## 3. Results

### 3.1. Subject Descriptive Characteristics

The subject’s descriptive characteristics are displayed in Table 1. On average, the subjects (*n* = 15) were middle-aged, overweight men who were fully active career firefighters. The mean values in Table 1 indicated they had “good” cardiorespiratory fitness and their waist circumference combined with their BMI placed them at “increased risk” for Type 2 Diabetes, hypertension, and CVD for men of their age [35]. They had normal lipid-lipoproteins and insulin, and elevated blood glucose levels [36]. The firefighters had elevated resting BP assessed by oscillometric methods in the clinic [19], while their 19 h ABP values classified them as having hypertension [37]. There were two subjects who were on antihypertensive medications, a beta-blocker and the other a diuretic. There were no smokers among the 15 subjects.

### 3.2. Nutrient Intake

The dietary intake of the subjects is depicted in Table 2. On average, the firefighters consumed below the dietary reference intake (DRI) for grams of fiber; milligrams of calcium, potassium, and magnesium; micrograms of vitamin D; total number of fruits, vegetables, and whole grain servings; ounces of lean meat from nuts/seeds; and total caloric intake [38]. They consumed above the DRI for grams of carbohydrates, protein, fat, and trans fatty acids, and milligrams of caffeine, cholesterol, and sodium. The firefighters were light drinkers based on their self-reported alcohol intake [38].

### 3.3. Dietary Approaches to Stop Hypertension

The DASH accordance score is presented in Table 3. On average, the firefighters’ overall DASH score was just below 3 points, indicating poor DASH accordance. Percent energy from total and saturated fat exceeded and average calcium, potassium, and fiber intake were below the DASH recommended levels scoring 0 points. Average sodium, cholesterol, and magnesium were within the intermediate target range of the DASH recommendations scoring 0.5 points. Percent energy from protein met the target value scoring 1 point. Of note, the DASH score was not correlated with the ABP response to strenuous physical exertion among our firefighters (*p*s > 0.05).

### 3.4. The Ambulatory Blood Pressure Response to Exercise

**Figure 2:**
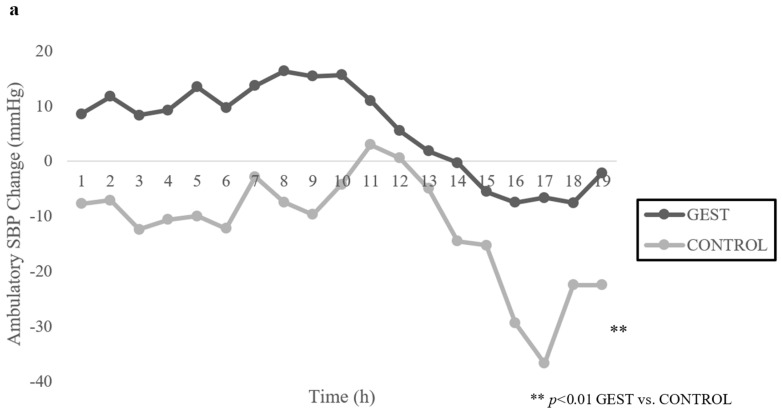

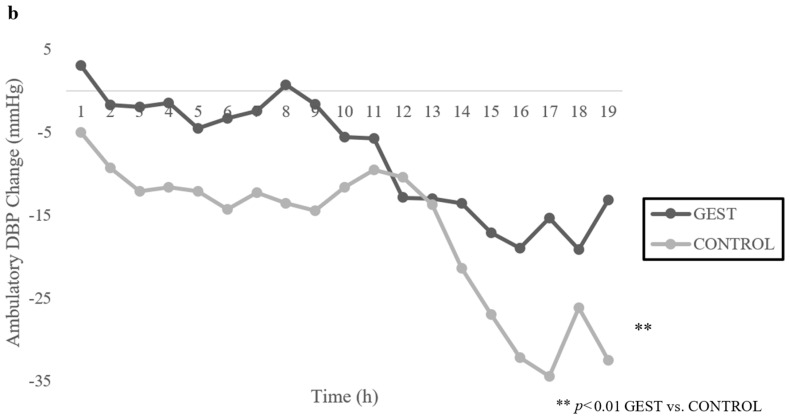
(**a**,**b**): Mean Change in Ambulatory Blood Pressure from Baseline Following GEST vs. CONTROL over 19 H (*n* = 15). Note: SBP = Systolic blood pressure, DBP = Diastolic blood pressure, GEST = Graded exercise stress test.

Figure 2a,b displays the ABP response following GEST and CONTROL over 19 h. Following the GEST, ambulatory SBP (ASBP) increased 6.4 ± 17.0 mmHg and ambulatory DBP (ADBP) decreased 7.3 ± 14.6 mmHg (*p*s < 0.01) over 19 h. Following CONTROL, ASBP decreased 11.6 ± 21.8 mmHg and ADBP decreased 16.4 ± 13.1 mmHg (*p*s < 0.01) over 19 h. Thus, ASBP was higher by 18.0 ± 6.7 mmHg and ADBP was higher by 9.1 ± 5.4 mmHg (*p*s < 0.01) following the GEST versus CONTROL over 19 h.

### 3.5. The Influence of Dietary Intake and the Cardiometabolic Biomarkers on the Ambulatory Blood Pressure Response to Exercise

Table 4 displays the linear mixed model findings for caffeine, calcium, and sodium and the cardiometabolic biomarkers that explained the largest portion of variance of the ABP response to exercise and whose variance inflation factors indicated there was minimal overlap among the independent variables. In addition, based on our empirical statistical power calculation, the power estimates indicated more than adequate power to test each independent variable and the overall model for statistical significance.

#### 3.5.1. Caffeine

Caffeine intake explained 23.6% (*p* = 0.0005) of the variance in the ASBP response to GEST versus CONTROL over 19 h; and when combined with post-GEST hs-CRP, baseline non-HDL-C, BMI, glucose, and resting heart rate, they explained 69.6% of the variance (*p* < 0.0001). Caffeine intake explained 11.1% (*p* < 0.0001) of the variance in the ADBP response to GEST versus CONTROL over 19 h; and when combined with baseline non-HDL-C, glucose, and BMI, they explained 74.2% of the variance (*p* < 0.0001).

#### 3.5.2. Calcium

Calcium intake explained 23.7% (*p* = 0.0005) and Vitamin D intake explained 28.5% (*p* = 0.0002) of the variance in the ASBP response to GEST versus CONTROL over 19 h; and when combined with resting heart rate and baseline fibrinogen, they explained 46.4% of the variance (*p* = 0.0009). Calcium intake explained 22.8% (*p* < 0.0001) and Vitamin D intake explained 7.0% (*p* = 0.0012) of the variance in the ADBP response to GEST versus CONTROL over 19 h; and when combined with resting DBP, baseline fibrinogen, and the ratio of total cholesterol to triglycerides, they explained 82.1% of the variance (*p* < 0.0001).

#### 3.5.3. Sodium

Sodium intake explained 5.0% (*p* = 0.0972) of the variance in the ASBP response to GEST versus CONTROL over 19 h not achieving statistical significance; but when combined with post-GEST hs-CRP, baseline non-HDL-C, and BMI, they explained 50.8% of the variance (*p* = 0.0003). Sodium intake explained 22.9% (*p* < 0.0001) of the variance in the ADBP response to GEST versus CONTROL over 19 h; and when combined with baseline cystatin C, BMI, resting DBP, and the ratio of total cholesterol to HDL-C, they explained 68.8% of the variance (*p* < 0.0001).

### 3.6. The Influence of Dietary Intake on Resting Blood Pressure

Habitual caffeine intake tended to be positively correlated with resting SBP (r = 0.498, *p* = 0.059) and DBP (r = 0.499, *p* = 0.058), explaining 25% of the individual variance in resting SBP and DBP. There were no significant correlations between calcium, vitamin D, and sodium intake and resting BP (*p*s > 0.05). The DASH score was positively correlated with resting SBP (r = 0.541, *p* = 0.037), explaining 29% of the variance in resting SBP. There were no significant correlations between the DASH score and resting DBP (*p* > 0.05).

The multivariate linear regression in Table 5 revealed the DASH score explained 2.2% (*p* = 0.245) of the variance in resting SBP not achieving statistical significance; but when combined with baseline non-HDL-C, post-GEST hs-CRP, and BMI, they explained 86.0% of the variance (*p* < 0.001).

## 4. Discussion

In this sub-study we conducted secondary statistical analyses from a larger clinical trial (NCT04514354) that included examining the relationship between habitual caffeine intake, and cardiometabolic biomarkers shown to influence BP [9,11,16,17,18], on the ABP response following a bout of sudden vigorous physical exertion among career firefighters. Other secondary aims were to explore the relationships between habitual calcium and sodium intake, and cardiometabolic biomarkers shown to influence BP [9,11,16,17,18], on the ABP response. Unexpectedly, rather than PEH, the firefighters exhibited postexercise hypertension, as ABP was higher by ~9–18 mmHg following GEST compared to CONTROL over 19 h. This excessive rise in ABP following maximal exertion is worrisome as it may contribute to the disproportionate number of acute cardiac events in firefighters [39]. Our study is the first to investigate the effects of habitual dietary intake on the ABP response to sudden vigorous exercise among firefighters. As we hypothesized, the firefighters’ dietary intake of caffeine, calcium, and sodium modulated the adverse ABP response to vigorous exertion with each factor individually explaining up to 23% to 24% of the ABP response.

### 4.1. Caffeine and the Cardiometabolic Biomarkers Associated with the ABP Response

Caffeine has beneficial effects on firefighter job performance as it aids in reducing fatigue and improves psychomotor vigilance [40,41]. The firefighters in our study consumed an average of 542 mg/d of caffeine, equivalent to ~6 cups of coffee, which is ~50% more than the DRI [38]. Their high caffeine intake accounted for ~24% of the adverse ASBP response and ~11% of the ADBP response after the GEST versus CONTROL over 19 h. Similarly, Notarius et al. found SBP was 9 mmHg higher following acute maximal aerobic exercise after receiving an intravenous caffeine infusion versus a placebo dextrose infusion in healthy adults with elevated resting BP [15]. Sarmento et al. found SBP was 11% and DBP was 18% higher from baseline following acute submaximal aerobic exercise after consuming caffeine versus water in subjects with controlled hypertension [14]. Notarius et al. and Sarmento et al. speculated excessive intake of caffeine had an adverse effect on the BP response to acute exercise due to inhibition of the vasodilatory actions of adenosine by attaching to its receptors, thereby promoting a vasoconstrictive response and acutely increasing BP [14,15].

We found in our linear mixed models that ~70% of the variance in the adverse ASBP and 74% of the variance in the ADBP response to maximal physical exertion was explained by caffeine intake, in addition to the cardiometabolic biomarkers of CRP, non-HDL-C, BMI, blood glucose, and resting heart rate. High CRP levels, a marker of systemic inflammation, which explained 44% of the ASBP variance, have been associated with an exaggerated SBP response during submaximal aerobic exercise attributed to attenuated nitric oxide production, a potent vasodilator [42,43]. Nonetheless, the firefighters in our study had normal CRP levels so it remains unclear how CRP was implicated in the adverse ASBP response we observed. Higher total blood cholesterol levels have been shown to inhibit the production of vasodilators produced by the endothelium contributing to an elevated BP response during an acute bout of submaximal aerobic exercise [44]. In our study, the firefighters had normal total cholesterol, but elevated non-HDL-C levels, which have emerged as a predictor of future cardiovascular events [45,46], and non-HDL-C explained up to 35% of the ASBP response and 45% of the ADBP response. Jarrett et al. found the onset of the BP lowering effects of exercise was delayed in men with obesity and hypertension [47], possibly due to over reactivity of the renin-angiotensin aldosterone and sympathetic nervous systems [48]. Moreover, Zeigler et al. concluded PEH was abolished among men with obesity and elevated BP [49]. In our study, the firefighters’ BMI explained 23% of the variance in the ASBP response and 32% of the ADBP response.

The firefighters in our study had elevated fasting blood glucose but normal insulin levels, the former of which explained 6% of the adverse ASBP and 38% of the ADBP response. We previously found among adults with elevated BP who were overweight to obese, a study population like the firefighters in this study, fasting blood glucose explained 6% of the peak SBP to a GEST. In that study, we speculated the presence of hyperglycemia attenuated nitric oxide production possibly contributing to an exaggerated peak SBP response to the GEST [50]. It appears that caffeine and the cardiometabolic biomarkers that emerged from our linear mixed models could have shifted the vasoactive balance of the endothelium towards vasoconstriction, contributing to the adverse ABP response to vigorous exercise we observed in the firefighters. Further investigation is needed to confirm our findings and better elucidate the regulatory interactions of habitual caffeine intake with these vasoactive cardiometabolic biomarkers and the ABP response to vigorous exercise.

### 4.2. Calcium and the Cardiometabolic Biomarkers Associated with the ABP Response

Adequate calcium intake prevents the development of hypertension [16]. The firefighters in our study consumed an average of 840 mg/d of calcium, equivalent to ~3 cups of 1% milk daily, which is ~15% less than the DRI [38]. Their calcium intake explained up to 24% of the adverse ABP response after the GEST compared to CONTROL over 19 h. Villa-Etchegoyen et al. speculated a calcium intake <1000 mg/d initiated the release of parathyroid hormone and activated the renin-angiotensin aldosterone system, leading to vasoconstriction and increased peripheral vascular resistance, thereby resulting in increased BP [51]. We previously found a calcium intake of 1380 mg/d equivalent to ~40% more than the DRI, contributed to ASBP being 6 mmHg higher after a bout of acute aerobic submaximal exercise than a calcium intake of 659 mg/d equivalent to 33% less than the DRI among 50 overweight men with hypertension [16]. The discrepancy between our earlier and current findings may be attributed to the methods used to assess calcium intake as we used three-day dietary recalls on five occasions in our earlier study and the current study assessed habitual calcium intake over the past year using a food-frequency questionnaire. Furthermore, the subjects in our earlier study performed acute bouts of submaximal aerobic exercise whereas the firefighters in the current study completed a maximal GEST.

We found in our linear mixed models that ~46% of the variance in the ASBP and ~82% of the variance in the ADBP response to vigorous exertion was explained by calcium and vitamin D intake, in addition to the cardiometabolic biomarkers of resting heart rate, fibrinogen, resting DBP, and the ratio of total cholesterol to triglycerides. Vitamin D explained 29% of the adverse ASBP response and 7% of the ADBP response. Our firefighters consumed insufficient levels of vitamin D (~4 mcg/d) that was ~70% less than the DRI. Forman et al. found individuals with insufficient vitamin D levels were three times more likely to develop future hypertension compared to those with sufficient levels [52]. Ni et al. and Schulze et al. found insufficient vitamin D levels shifted the vasoactive balance of the endothelium towards vasoconstriction, possibly partially explaining the adverse ABP response among firefighters [53,54]. Fibrinogen explained 14% of the variance in the ASBP response and 31% of the ADBP response among the firefighters. Shankar et al. found that elevated fibrinogen levels, a predictor of CVD, had a positive association with 5-year incident hypertension among 286 men who were overweight with elevated BP possibly due to increased blood viscosity, peripheral vascular resistance, and insulin resistance [55]. It is well established the BP response to exercise is a function of resting BP [9,11,12,56,57]. Our sample had elevated resting DBP which explained 58% of the adverse ADBP response. Like habitual caffeine intake, habitual calcium and vitamin D intake and their interactions with the cardiometabolic biomarkers that emerged in the statistical models may have shifted the vasoactive balance of the endothelium towards vasoconstriction, contributing to the adverse ABP response to maximal physical exertion we observed in the firefighters.

### 4.3. Sodium and the Cardiometabolic Biomarkers Associated with the ABP Response

The firefighters in our study consumed an average of 2643 mg/d of sodium, equivalent to ~1.1 teaspoons of salt, which is ~75% more than the DRI [38]. Their high sodium intake explained ~23% of the ADBP response after the GEST versus CONTROL over 19 h. Consistent with our findings, Babcock et al. conducted a randomized crossover trial among 19 healthy subjects with normal to elevated BP who consumed either a sodium capsule containing 3900 mg of sodium for 10 days, equivalent to 158% more than the DRI, or a placebo capsule of dextrose for 10 days. The high intake of sodium resulted in SBP being 8 mmHg higher following acute submaximal aerobic exercise than the placebo experiment [17]. Furthermore, the high sodium intake reduced flow-mediated dilation compared to placebo. Babcock et al. concluded 10 days of high sodium intake impaired endothelial function, which then in turn adversely impacted the SBP response to an acute bout of submaximal exercise [17].

Among our firefighters, habitual sodium intake was a significant predictor only in the ADBP linear mixed model explaining 23% of the variance. We found a total of ~69% of the variance in the ADBP response to vigorous exertion was explained by sodium intake, in addition to the cardiometabolic biomarkers of cystatin C, BMI, resting DBP, and the ratio of total cholesterol to HDL-C. Kestenbaum et al. found for each 15-nmol/L increase in cystatin C, incident hypertension increased by 15% among overweight adults with normal BP [58]. The firefighters had normal cystatin C levels so it is unclear how cystatin C would contribute to the adverse ADBP response we observed. BMI explained ~26% of the adverse ADBP response. As stated previously, the onset of the BP lowering effects of exercise were delayed or absent in men with obesity and hypertension [47,49]. Overweight and obesity increase renal sodium reabsorption and activate the renin-angiotensin and sympathetic nervous systems which elevate BP [59]. Resting DBP explained ~12% of the ADBP response. As stated previously, the BP response to exercise is a direct positive function of resting BP [9,11,12,56,57]. The firefighters had a normal total cholesterol to HDL-C ratio, but elevated non-HDL-C levels which is an emerging CVD risk factor [45,46]. Additional research is needed to better ascertain the mechanisms by which the ABP response to vigorous physical exertion among the firefighters is adversely affected by high habitual sodium and caffeine and low dietary calcium intake as they interact with the vasoactive biomarkers that emerged from our linear mixed models.

### 4.4. Dietary Approaches to Stop Hypertension

Because of the favorable influence of the DASH diet on hypertension [26,27], it is important to note the firefighters in our sub-study had a below average DASH accordance score. Yet, the DASH score was not significantly associated with resting SBP nor the ABP response to strenuous physical exertion among our firefighters.

### 4.5. Limitations and Strengths

Our study is a secondary analysis of a larger clinical trial among a small sample of overweight Caucasian male career firefighters, so the generalizability of our findings is restricted. However, our sample was representative of the United States firefighting population as 96% are male [60] and >70% are overweight or obese [1]. We used a maximal GEST to simulate firefighting demands, which does not adequately replicate the extreme physical and mental demands of the job. The firefighters self-reported habitual dietary intake over the past year with the NHANES Food-Frequency Questionnaire [22,23,24,25] for which underreporting has been seen in overweight to obese populations. Furthermore, the accuracy of reporting may be affected by utilizing a one-year time frame of recall [61]. Nonetheless, this time frame reflects habitual dietary intake, which may be more related to BP than a shorter time frame of recall. Our study had several strengths. The order of the two experiments was randomized to account for the circadian variation in ABP and isolate the influence of the GEST from CONTROL on the ABP response to exercise. We used the clinical gold standard of ABP monitoring and minimized technical error by having all BP assessments performed by a single investigator at the same time of day using the same ABP monitor for the same subject throughout the study.

## 5. Conclusions

To the best of our knowledge, this is the first randomized controlled clinical trial to analyze the relationship between habitual dietary intake on the ABP response in firefighters, a recommended research area to improve firefighter health and safety in the 2021 National Fire Service Research Agenda Report [8]. Surprisingly, the firefighters exhibited postexercise hypertension rather than PEH. This adverse ABP response was partially attributed to the habitual intake of caffeine, calcium, and sodium with each accounting for up to 24% of the variance in the adverse ABP response. Our linear mixed models revealed these dietary factors combined with vasoactive cardiometabolic biomarkers explained a clinically meaningful proportion of the variance in the adverse ABP response. Further investigation is needed in a larger sample of more diverse firefighters to better establish the influence that these dietary factors and their interactions with cardiometabolic biomarkers have on their adverse ABP response to vigorous exertion. If our findings are confirmed, lifestyle interventions will need to be implemented to improve the diet and CVD risk factor profile of firefighters.

## Figures and Tables

**Table 1:** Subject Characteristics (Mean ± SD).

	Total Sample (*n* = 15)
Age (yr)	40.2 ± 9.5
BP medication use (Yes/No)	2/13
DASH accordance score	2.8 ± 1.3
BMI (kg/m^2^)	29.0 ± 3.9
Waist Circumference (cm)	93.3 ± 11.1
Resting SBP (mmHg)	124.1 ± 10.3
Resting DBP (mmHg)	79.6 ± 11.5
19 h Ambulatory SBP (mmHg)	134.8 ± 7.4
19 h Ambulatory DBP (mmHg)	76.6 ± 4.7
Total Cholesterol (mg/dL)	186.7 ± 33.0
LDL-C (mg/dL)	114.5 ± 30.1
HDL-C (mg/dL)	48.4 ± 11.4
Triglycerides (mg/dL)	118.3 ± 86.8
Non-HDL-C (mg/dL)	138.3 ± 37.8
Total Cholesterol/Triglycerides (U)	2.1 ± 0.8
Total Cholesterol/HDL-C (U)	4.1 ± 1.5
Glucose (mg/dL)	98.7 ± 16.9
Insulin (ulU/mL)	6.5 ± 4.1
Relative peak oxygen consumption (mL/kg·min^−1^)	41.5 ± 6.8

Note: BP = Blood pressure, DASH = Dietary Approaches to Stop Hypertension, BMI = Body mass index, SBP = Systolic blood pressure, DBP = Diastolic blood pressure, LDL-C = Low-density lipoprotein-cholesterol, HDL-C = High-density lipoprotein-cholesterol.

**Table 2:** Daily Average Nutrient Intake (mean ± SD) Compared to the Dietary Reference Intake.

	Daily Intake (*n* = 15)	Dietary Reference Intake
Carbohydrate (g)	171.9 ± 64.8	130
Protein (g)	80.0 ± 30.4	56
Fat (g)	68.3 ± 28.5	56
Fiber (g)	14.1 ± 6.0	38
Cholesterol (mg)	222.4 ± 84.5	<200
Vitamin D—Calciferol (mcg)	4.1 ± 2.6	15
Caffeine (mg)	542.0 ± 348.9	400
Calcium (mg)	840.7 ± 369.8	1000
Potassium (mg)	3070.5 ± 971.5	4700
Magnesium (mg)	331.6 ± 116.4	420
Sodium (mg)	2642.9 ± 1004.9	1500
Trans Fatty Acids (g)	3.0 ± 1.1	2
Total Number of Fruit Servings	1.7 ± 1.1	4
Total Number of Vegetable Servings	2.8 ± 1.3	5
Lean Meat Equivalent from Nuts/Seeds (Oz)	0.5 ± 0.5	0.7
Lean Meat from Fish/Other Seafood (Oz)	0.5 ± 0.5	0.3–0.4
Number of Whole Grain Servings	0.7 ± 0.4	3
Alcohol (g)	12.9 ± 10.5	≤28
Total Caloric Intake (kcal)	1683.2 ± 621.4	2538

Note: Dietary Reference Intake values retrieved from: National Institutes of Health. Nutrient Recommendations: Dietary Reference Intakes (DRI). 2020, https://ods.od.nih.gov/HealthInformation/nutrientrecommendations.aspx, accessed on 7 August 2020 [38].

**Table 3:** DASH Diet Nutritional Intake (mean ± SD) and Nutrient Targets.

	Total Sample Intake (*n* = 15)	DASH Score Target (1 Point)	DASH Score Intermediate Target (0.5 Points)	DASH Score Not Meeting Target or Intermediate (0 Points)
DASH accordance score	2.8 ± 1.3			
DASH nutrients				
Sodium (mg/d)	2642.9 ± 1001.9	<2300.0	2300–2650 ^b^	>2650
Cholesterol (mg/d)	222.4 ± 84.5	<149.1	149.1–224.7 ^b^	>224.7
Saturated fat (% of kcal/d)	11.6 ± 2.6	<6.0	6–11	>11 ^c^
Total fat (% of kcal/d)	36.5 ± 5.0	<27.0	27–32	>32 ^c^
Protein (% of kcal/d)	19.2 ± 2.6	>18.0 ^a^	16.5–18.0	<16.5
Calcium (mg/d)	840.7 ± 369.8	>1240.0	842.3–1240.0	<842.3 ^c^
Magnesium (mg/d)	331.6 ± 116.4	>496.7	330.3–496.7 ^b^	<330.3
Potassium (mg/d)	3070.5 ± 971.5	>4673.3	3198.3–4673.3	<3198.3 ^c^
Fiber (g/d)	14.1 ± 6.0	>30.0	19.5–30.0	<19.5 ^c^

Note: DASH = Dietary Approaches to Stop Hypertension; ^a^ = 1 point, ^b^ = 0.5 points, ^c^ = 0 points.

**Table 4:** The Proportion of Variance Explained in the Linear Mixed Models for the ASBP and ADBP Response to GEST vs. CONTROL over 19 h.

Predictors	*Β*	T	Partial *PVE*	VIF	Power
Caffeine					
ASBP			0.696 † *		1.000 ^¶^
C-reactive Protein	−8.75	−8.78	0.444 *	4.91486	1.000
Non-HDL-C	−0.22	−7.44	0.346 *	1.95318	1.000
Caffeine	0.02	5.66	0.236 ^c^	2.63521	0.991
Body Mass Index	1.97	5.80	0.228 ^c^	3.06201	0.994
Glucose	0.18	2.80	0.062 ^a^	2.09854	0.771
Heart Rate	−0.17	−1.96	0.033 ^#^	1.58699	0.610
ADBP			0.742 † *		1.000 ^¶^
Non-HDL-C	−0.29	−67.58	0.446 *	1.29845	1.000
Glucose	−0.11	−17.17	0.375 *	1.20414	1.000
Body Mass Index	0.51	14.58	0.315 *	1.20949	1.000
Caffeine	−0.01	−13.52	0.111 *	1.11281	1.000
Calcium					
ASBP			0.464 † ^c^		1.000 ^¶^
Vitamin D	4.24	5.54	0.285 ^c^	1.59214	0.998
Calcium	−0.02	−5.04	0.237 ^c^	1.62399	0.992
Heart Rate	−0.43	−3.76	0.192 ^b^	1.09861	0.954
Fibrinogen	0.09	2.93	0.143 ^a^	1.08374	0.888
ADBP			0.821 † *		1.000 ^¶^
Resting DBP	−0.72	−48.00	0.582 *	1.35211	1.000
Fibrinogen	0.08	14.81	0.309 *	1.16022	1.000
Calcium	−0.01	−12.28	0.228 *	1.83184	1.000
TC/TG	−2.34	−5.34	0.093 ^c^	1.23754	0.984
Vitamin D	0.79	4.68	0.070 ^b^	1.76900	1.000
Sodium					
ASBP			0.508 † ^c^		1.000 ^¶^
C-reactive Protein	−4.91	−4.97	0.302 ^c^	1.84193	0.966
Non-HDL-C	−0.16	−4.27	0.234 ^b^	1.51272	0.925
Body Mass Index	1.02	2.33	0.068 ^a^	2.55082	0.619
Sodium	−0.00	−1.83	0.050 ^#^	1.11441	0.507
ADBP			0.688 † *		1.000 ^¶^
Cystatin C	46.81	23.18	0.270 *	2.90843	1.000
Body Mass Index	1.37	17.62	0.263 *	1.64401	1.000
Sodium	0.01	19.86	0.229 *	2.77005	1.000
Resting DBP	−0.20	−8.83	0.118 *	2.16846	1.000
TC/HDL-C	−7.01	−11.24	0.095 *	1.77217	1.000

Note: ASBP = Ambulatory systolic blood pressure, ADBP = Ambulatory diastolic blood pressure, GEST = Graded exercise stress test, PVE = Portion of variance explained, VIF = Variance inflation factor, HDL-C = High-density lipoprotein-cholesterol, DBP = Diastolic blood pressure, TC = Total cholesterol, TG = Triglycerides; † Total variance explained of the model (PVE), ^¶^ Post hoc statistical power of the model, ^#^ = *p* > 0.05, ^a^ = *p* < 0.05, ^b^ = *p* < 0.01, ^c^ = *p* < 0.001, * = *p* < 0.0001.

**Table 5:** The Proportion of Variance Explained in the Multivariate Linear Regression Models for Resting Blood Pressure.

Predictors	*Β*	T	Partial *PVE*	VIF
SBP			0.860 † ^b^	
Non-HDL-C	0.68	5.00	0.351 ^a^	1.332
C-reactive Protein	0.56	3.85	0.209 ^a^	1.490
Body Mass Index	−0.33	−2.17	0.066 ^#^	1.635
DASH Score	−0.18	−1.23	0.022 ^#^	1.470

Note: PVE = Portion of variance explained, VIF = Variance inflation factor, SBP = Systolic blood pressure, HDL-C = High-density lipoprotein-cholesterol, DASH = Dietary Approaches to Stop Hypertension; † Total variance explained of the model (PVE), ^#^ = *p* > 0.05, ^a^ = *p* < 0.01, ^b^ = *p* < 0.001.

## Data Availability

The data presented in this study will be made available upon reasonable request to the corresponding author.
